# Angiosarcoma of the abdominal aorta after previous endovascular aneurysm repair

**DOI:** 10.1016/j.jvscit.2025.101878

**Published:** 2025-06-13

**Authors:** Ghulamhadi Momand, Maarten J. van der Laan, Clark J. Zeebregts, Ignace F.J. Tielliu

**Affiliations:** Department of Surgery (Division of Vascular Surgery), University Medical Center Groningen, University of Groningen, Groningen, The Netherlands

**Keywords:** Aneurysm, EVAR, Angiosarcoma

## Abstract

Primary aortic angiosarcoma is a rare and aggressive malignancy arising from endothelial cells of blood vessels and has a poor prognosis. We report 21 relevant cases from the literature, summarizing the number of reported cases without analyzing treatment outcomes or prognostic data. Additionally, we report our own case of a 75-year-old male who presented with fever, abdominal pain, and leg discomfort 10 years after endovascular aneurysm repair (EVAR) for an abdominal aortic aneurysm. Initial imaging suggested an infected EVAR, leading to explantation and reconstruction. Despite antibiotic treatment, negative cultures and surgical intervention, the patient’s condition continued to deteriorate. A biopsy of cervical lymph nodes revealed a high-grade angiosarcoma. These cases highlight the diagnostic challenges of primary aortic angiosarcoma, especially in patients with prior EVAR, and emphasize the importance of considering rare malignancies in cases when a patient deteriorates despite therapy or does not show the expected treatment response.

Primary aortic angiosarcomas, originating from the intimal wall of the aorta, are rare and represent approximately 4.7% of all angiosarcomas.^1^^,^^2^ A recent systematic review reported 123 cases of primary aortic angiosarcomas.^2^ Primary aortic angiosarcomas occurring after endovascular aneurysm repair (EVAR) are even rarer, with only a few patient cases published. The rarity of the condition may contribute to delayed diagnosis. We present a patient case in which infection of an endoprosthesis after aneurysm repair was initially suspected, but further pathologic examination revealed the presence of an angiosarcoma.

## Case report

**Fig 1:**
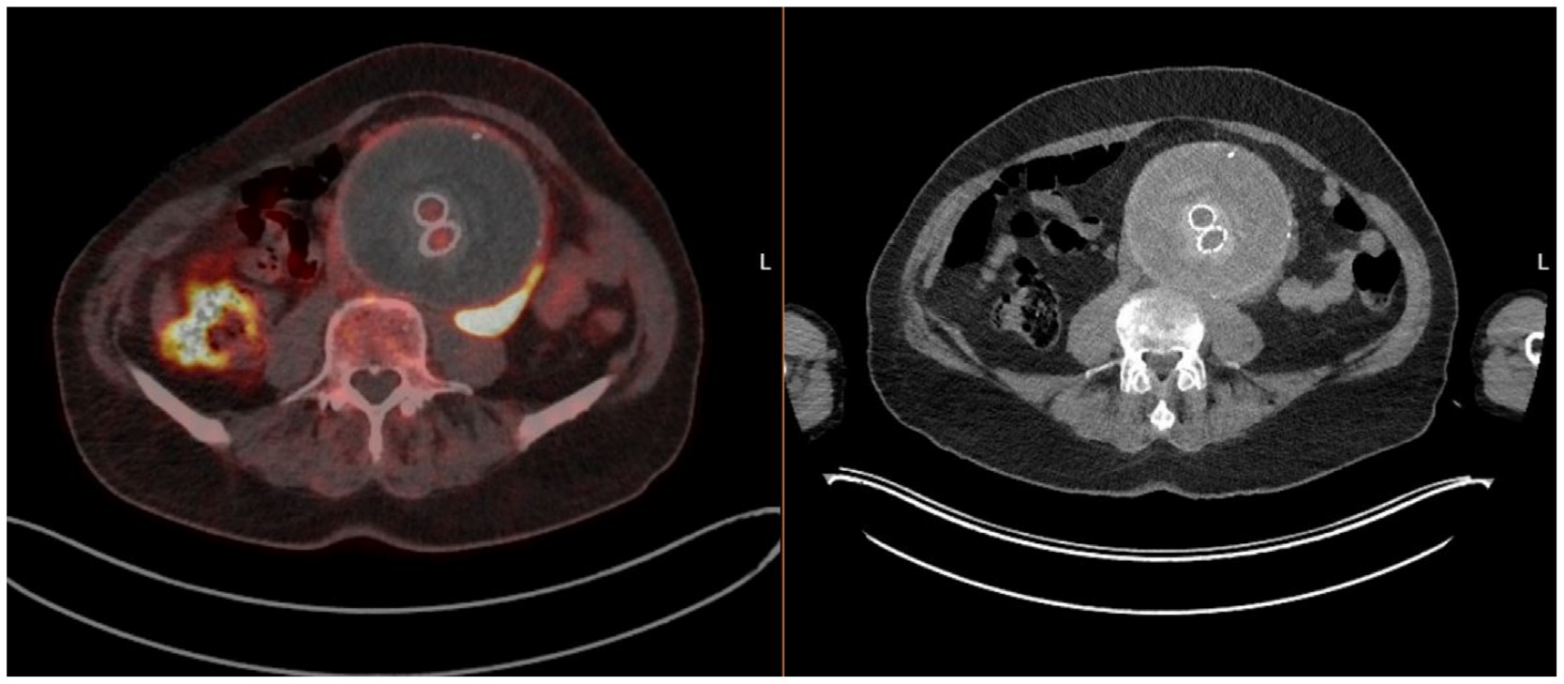
Initial positron emission tomography (PET)-computed tomography (CT) in the referring hospital showing fluid visible on the left lateral side of the aneurysm sac and increased ^18^fluorodeoxyglucose (FDG) uptake.

A 75-year-old male patient was referred to our hospital with suspected infection of an endoprosthesis (Gore Excluder, W.L. Gore & Associates), which was previously placed in 2013 to treat an infrarenal abdominal aortic aneurysm. That procedure was uneventful. The patient took acetylsalicylic acid 80 mg once daily. In April 2023, he presented to a referral hospital with abdominal pain, pain in the left leg, a feverish sensation, and general physical deterioration. Hemoglobin level was 6.6 mmol/L, leukocyte count was 11.3 × 10^9^/L, and C-reactive protein (CRP) was 59 mg/L. An ^18^fluorodeoxyglucose (FDG) positron emission tomography (PET)-computerized tomography angiography (CTA) scan showed uptake suspicious for infection at the site of a fluid collection on the left lateral side of the aneurysm sac (Fig 1). Several blood cultures were negative. A computed tomography (CT)-guided puncture of the fluid collection was performed, and although the cultures were negative, a positive 16S polymerase chain reaction (PCR) analysis of the punctured fluid was obtained, showing predominantly streptococci and staphylococci. The bacterial load was very low (cycle threshold value of 40), making it unclear whether the PCR result represented either an actual pathogen or contamination. In November 2023, the patient was admitted to our center. Broad-spectrum antibiotics (piperacillin/tazobactam, vancomycin, and caspofungin) were initiated in accordance with our vascular prosthetic infection protocol. Surgery seemed indicated due to a suspected infection, supported by imaging findings (increased FDG uptake around the aneurysm), elevated CRP and leukocyte levels, and clinical symptoms consistent with infection including fatigue, fever, and abdominal pain, as well as a positive 16S PCR for *Streptococcus* and *Staphylococcus*, although the bacterial load was very low. The infected EVAR was explanted, and a neo-bifurcation was constructed using an autologous superficial femoral vein from the right leg and connecting the infrarenal aorta to the left and right common iliac arteries. Pathologic examination of the aortic wall showed a transmural chronic lymphocytic inflammatory cell infiltrate without significant evidence of IgG4-mediated disease. A colony of Corynebacterium glucuronolyticum was isolated from the fluid collection near the aneurysm. However, this was not considered definitive evidence of a vascular prosthetic infection. In several multidisciplinary team meetings, it was decided to discontinue antibiotics 2 weeks after explantation of the EVAR.

**Fig 2:**
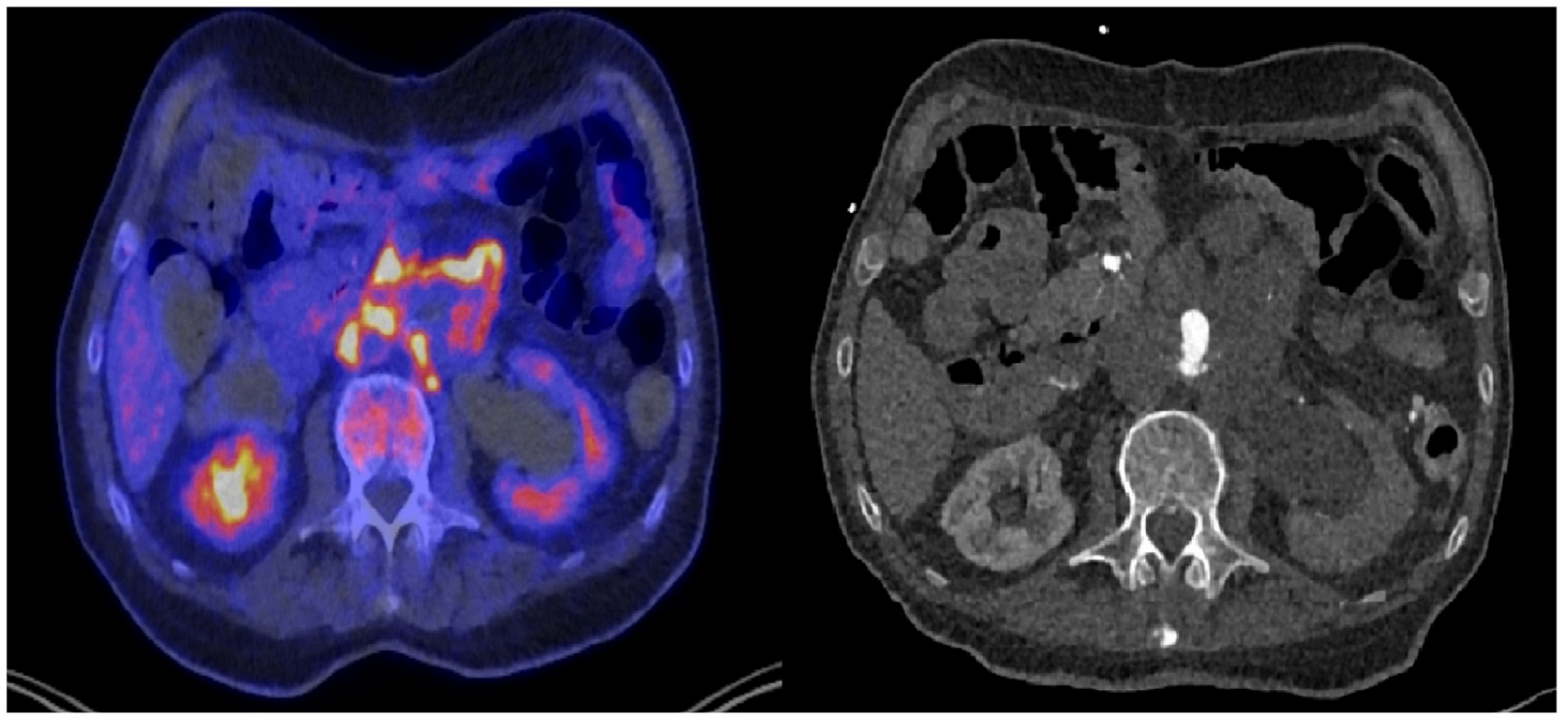
Positron emission tomography (PET)-computed tomography (CT) scan showing ^18^fluorodeoxyglucose (FDG) uptake around the venous reconstruction of the abdominal aorta.

**Fig 3:**
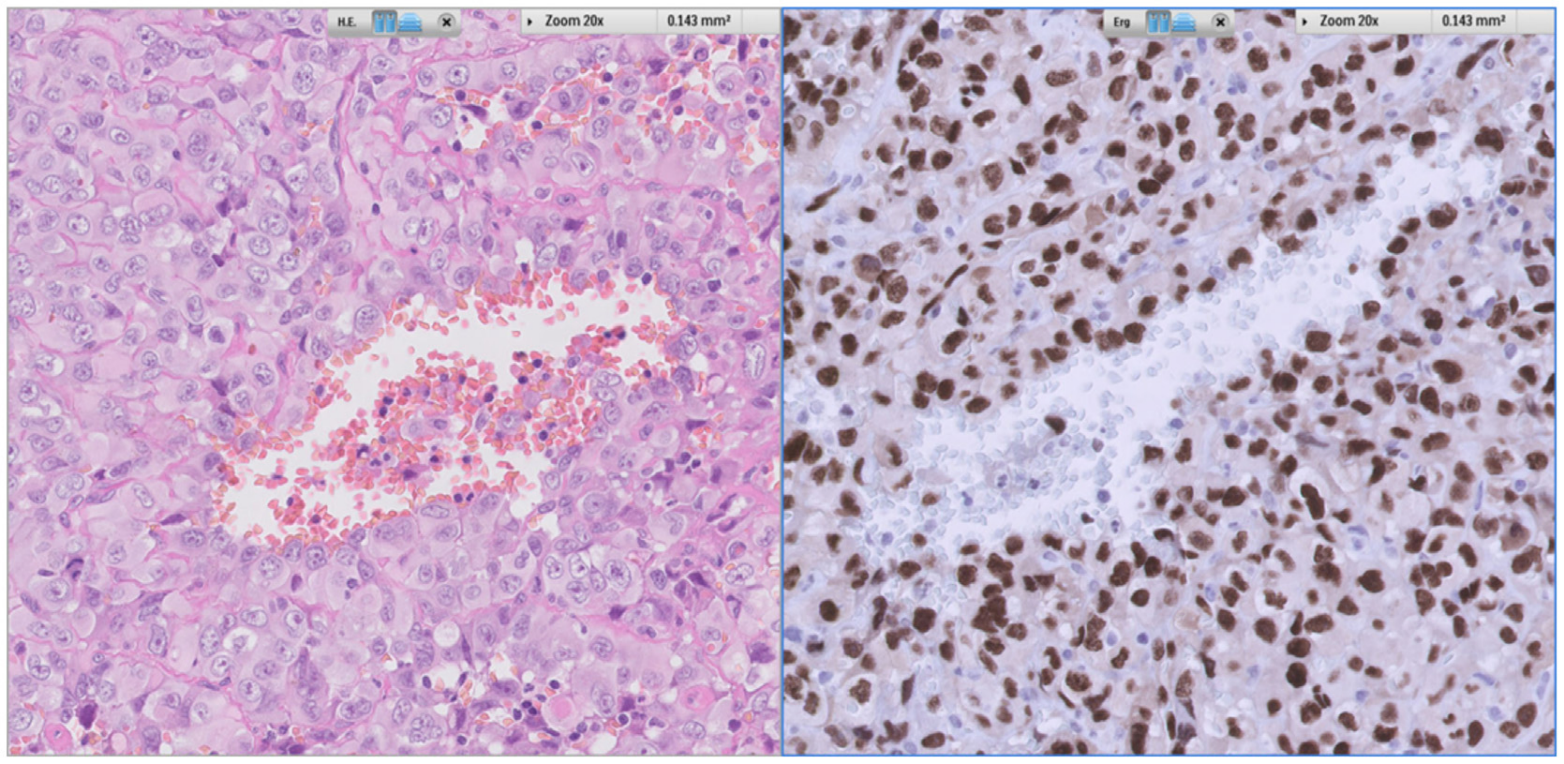
Pathologic examination of the lymph node: vascular marker ERG (endothelial receptor tyrosine kinase) and hematoxylin and eosin stain.

In March 2024, the patient was readmitted due to abdominal pain radiating to the shoulders and an elevated CRP level (93 mg/L). Imaging showed that the aneurysm sac had increased in size to 6 cm and was filled with fluid. The Hounsfield Unit value was 59, consistent with clotted blood. There were no signs of active bleeding. A CT-guided puncture of the fluid collection was performed, and a drain was left in place, producing 300 mL of bloody discharge per day. An ^18^FDG-PET-CT scan demonstrated FDG uptake around the venous reconstruction of the abdominal aorta (Fig 2), as well as hypermetabolic lymph nodes in the neck and foci in the right lung and liver. An infected venous reconstruction was suspected. The drain continued to produce a significant amount of blood, for which multiple blood transfusions were administered. However, no source of bleeding was identified on CTA and subsequent surgical exploration. The fluid collection around the aneurysm sac was drained during surgery, and macroscopically atypical lymph nodes were taken for histologic analysis. The cultures remained negative, and no malignancy was found in the perioperatively obtained tissue sent for pathologic examination, nor in the drain fluid or ultrasound-guided biopsy of the lymph node on the left side of the neck. Later, a lymph node in the neck was resected for diagnostic purposes, revealing a high-grade angiosarcoma on pathologic examination (Fig 3). The patient developed melena and low hemoglobin levels approximately 1 month after the surgical exploration. Gastroscopy revealed findings suspicious for an aorta-duodenal fistula, although the CTA was not suspicious for a fistula with a considerable distance between the vascular prosthesis and the duodenum. Due to the patient’s overall poor condition, a conservative treatment approach was decided. The patient died 4 days after discharge.

Given that the patient was deceased, written informed consent was obtained from the family.

## Discussion

Sarcomas are rare tumors that arise from the malignant transformation of cells in connective tissues, such as bone and supporting soft tissue. Angiosarcoma is a rare subtype of soft tissue sarcoma, characterized by the malignant transformation of endothelial cells that line the blood vessels or lymphatic channels. Angiosarcomas primarily develop in the skin and subcutaneous tissues (approximately 60%-70% of cases), but have also been observed in deeper soft tissues, such as the breast, lungs, spleen, liver, bone, and retroperitoneum. The hallmark of angiosarcoma is the presence of abnormal, pleomorphic, and malignant endothelial cells, which may have various shapes, including rounded, fusiform, epithelioid, or polygonal forms. Angiosarcomas are more commonly observed in older patients, with a mean age at diagnosis of 60 years, and occur with equal frequency in both sexes. It is estimated that angiosarcomas make up 5.4% of cutaneous soft tissue sarcomas and 2% of soft tissue sarcomas. The annual incidence of angiosarcoma is one case per million person.^3^, ^4^, ^5^ The abdominal aorta is the most common site of occurrence for angiosarcoma of the aorta.^2^^,^^6^ Patients typically present with intraarterial thromboembolic complications or general symptoms such as weight loss, fatigue, and fever.^3^^,^^7^, ^8^, ^9^ An EVAR stent may prevent the intraluminal proliferation of the tumor, thereby halting thromboembolic complications of the angiosarcoma, leading to patients presenting more frequently with nonspecific systemic complaints.^10^ EVAR is the preferred technique for the treatment of aortic aneurysms, but complications do occur including infection of the EVAR with reported incidence rates ranging from 0.2% to 0.7%.^11^ An infected EVAR can present with symptoms such as fatigue, weight loss, pain, and fever,^12^ symptoms that are also commonly observed in patients with aortic angiosarcomas. On imaging, the morphologic changes induced by an angiosarcoma, such as aortic wall irregularity, thrombus formation, an appearance sometimes that may mimic protrusive vegetation, and higher FDG uptake on PET scans, can be seen. These findings of aortic wall irregularity, higher FDG uptake, and protrusive appearance can make the image appear infectious in nature, leading to a diagnostic challenge.^2^^,^^4^^,^^13^, ^14^, ^15^ This may result in misdiagnosis and may complicate the identification of such a rare condition as an angiosarcoma. Initial imaging in the present case also suggested an infected EVAR. A focused literature search was performed using PubMed with the terms ‘angiosarcoma,’ ‘epithelioid sarcoma,’ ‘EVAR,’ and ‘endovascular repair.’ Articles were screened based on title and abstract, and reference lists were reviewed for relevant additional cases. We did not conduct a formal systematic review with Preferred Reporting Items for Systematic reviews and Meta-Analyses (PRISMA) methodology. This revealed 21 documented cases of aortic angiosarcomas arising after EVAR.^8^^,^^10^^,^^16^, ^17^, ^18^, ^19^, ^20^, ^21^, ^22^, ^23^, ^24^, ^25^, ^26^, ^27^, ^28^, ^29^, ^30^ In 13 of these cases, the initial clinical impression was that of an infected EVAR.^8^^,^^10^^,^^16^^,^^19^, ^20^, ^21^, ^22^, ^23^, ^24^^,^^26^^,^^28^^,^^30^ This further underscores the importance of considering rare differential diagnoses, such as angiosarcoma, in patients presenting with systemic complaints after EVAR. The prognosis for primary aortic tumors, including angiosarcomas, is poor. The 5-year survival rate is only 8%. These tumors are aggressive in nature and frequently metastasize, with a metastatic rate of 69% to 83% at the time of diagnosis. They mostly metastasize to the bones, lungs, liver, kidney, and peritoneum.^6^^,^^15^^,^^31^ This highlights the critical importance of early diagnosis, as prompt recognition and intervention could potentially improve the patient’s prognosis. Several small studies in the literature recommend radical surgical resection. Fatima et al^32^ presented a group of 13 patients in which the best survival outcomes were observed with a combination of surgical resection, aortic reconstruction, and chemoradiotherapy. However, early diagnosis of this disease remains challenging due to the rarity of the condition. In our case, a PET scan performed at the referring hospital in June 2023 already showed newly FDG-avid supraclavicular lymph nodes. A follow-up PET scan in October revealed an increase in the FDG-avid supraclavicular lymph nodes and a paratracheal lymph node on the left side, measuring 11 mm (previously 6 mm) with high FDG uptake. Initially, these findings were thought to represent reactive lymph nodes in response to the infected prosthesis. However, with the benefit of hindsight, an earlier biopsy of the lymph nodes might have been warranted, given their FDG uptake and progressive enlargement on imaging. This could have led to an earlier diagnosis and potentially prevented the surgery, considering the presence of metastases. It is unclear why the pathology of the aortic wall at the time of EVAR explant showed no angiosarcoma. It is possible that the angiosarcoma was still locally confined at the time of the EVAR explant, and the tissue sample submitted for histopathologic examination may not have been representative, thus not capturing the malignant component. The patient underwent five CTA scans following the EVAR procedure, prior to the clinical suspicion of prosthetic infection. Studies have reported an increased cancer risk associated with cumulative radiation exposure, particularly from long-term CTA follow-up after EVAR.^33^ In our case, it is conceivable that the repeated CTA scans contributed to the development of the angiosarcoma. However, we found no evidence in the literature linking this specifically to primary aortic angiosarcoma. Thus, although a contributory role is conceivable, a direct causal link remains speculative. The patient had no familial history of cancer.

## Declaration of generative AI and AI-assisted technologies in the writing process

During the preparation of this work, the author(s) used ChatGPT, OpenAI to check grammar/improve language. After using this tool/service, the author(s) reviewed and edited the content as needed and take(s) full responsibility for the content of the publication.

## Funding

None.

## Disclosures

None.
